# Therapeutic potential of silver nanoparticles from *Helianthemum lippii* extract for mitigating cadmium-induced hepatotoxicity: liver function parameters, oxidative stress, and histopathology in wistar rats

**DOI:** 10.3389/fbioe.2024.1400542

**Published:** 2024-06-27

**Authors:** Ibtissam Laib, Boutlilis Djahra Ali, Ali Alsalme, David Croun, Mikhael Bechelany, Ahmed Barhoum

**Affiliations:** ^1^ Department of Cellular and Molecular Biology, Faculty of Natural and Life Sciences, El Oued University, El Oued, Algeria; ^2^ Higher School of Saharan Agriculture, El Oued, Algeria; ^3^ Laboratory of Biology, Environment and Health, Faculty of Natural and Life Sciences, El Oued University, El-Oued, Algeria; ^4^ Department of Chemistry, College of Science, King Saud University, Riyadh, Saudi Arabia; ^5^ Institut Européen des Membranes, IEM, UMR-5635, University Montpellier, ENSCM, CNRS, Place Eugene Bataillon, Montpellier, France; ^6^ Gulf University for Science and Technology, GUST, Helwan, Kuwait; ^7^ NanoStruc Research Group, Chemistry Department, Faculty of Science, Helwan University, Cairo, Egypt

**Keywords:** silver nanoparticles, green synthesis, cadmium toxicity, hepatoprotective, wistar rats, biochemical parameters, oxidative stress, histopathology

## Abstract

**Introduction:** This study explores the therapeutic potential of silver nanoparticles (Ag NPs) synthesized using a *Helianthemum lippii* extract in mitigating cadmium-induced hepatotoxicity in Wistar rats. Given the increasing environmental and health concerns associated with cadmium exposure, novel and eco-friendly therapeutic strategies are essential.

**Methods:** Ag NPs were characterized using X-ray diffraction, UV-Vis spectrometry, and energy-dispersive X-ray spectroscopy with scanning electron microscopy, confirming their formation with a cubic crystal structure and particle sizes ranging from 4.81 to 12.84 nm. A sub-acute toxicity study of Ag NPs (2 mg/kg and 10 mg/kg) was conducted, showing no significant difference compared to untreated control rats (*n* = 3 animals/group). Subsequently, adult Wistar rats (*n* = 5/group) were divided into a control group and three experimental groups: Ag NPs alone, exposure to 50 mg/kg CdCl_2_ in drinking water for 35 days, and CdCl_2_ exposure followed by 0.1 mg/kg/day Ag NPs intraperitoneally for 15 days.

**Results:** In the CdCl_2_-exposed group, there was a significant decrease in body weight and increases in alanine and aspartate transaminase levels (*p* < 0.05 vs. control), indicating hepatotoxicity. Additionally, antioxidant defenses were decreased, and malondialdehyde levels were elevated. Liver histology revealed portal fibrosis, inflammation, necrosis, sinusoid and hepatic vein dilation, and cytoplasmic vacuolations. Treatment with Ag NPs post-CdCl_2_ exposure mitigated several adverse effects on liver function and architecture and improved body weight.

**Discussion:** This study demonstrates the efficacy of Ag NPs synthesized via a green method in reducing cadmium-induced liver damage. These findings support the potential of Ag NPs in therapeutic applications and highlight the importance of sustainable and eco-friendly nanoparticle synthesis methods. By addressing both toxicity concerns and therapeutic efficacy, this research aligns with the growing emphasis on environmentally conscious practices in scientific research and healthcare.

## 1 Introduction

Due to the rapid industrialization and the global population increase, environmental pollutants, particularly heavy metals, pose substantial health risks (Ye et al., 2009). Cadmium, is an exceptionally detrimental heavy metal widely present in soil, air, water, and various food sources, underscoring its pervasive environmental threat (Genchi et al., 2020). Extensive documentation highlights the effects of acute and chronic cadmium exposure on vital organs, such as liver and kidneys, emphasizing the critical need to comprehensively understand the associated health risks. The ultrastructural changes in these organs correlate with the exposure duration and type, pinpointing hepatocytes and endothelial cells as primary cell targets. Despite notable advances, more research is needed to comprehensively determine the implications of cadmium exposure (Al-Khayri et al., 2023). Investigations into the mechanisms of cadmium-induced acute hepatotoxicity unveiled two distinct pathways: initial damage caused directly by cadmium effects and subsequent damage triggered by inflammation (Li et al., 2023). The primary source of harm is due to Cd^+2^ binding to sulfhydryl groups in crucial mitochondrial components, leading to oxidative stress, altered mitochondrial permeability, and functional impairments (Peng et al., 2016). This shows the complex molecular pathways through which cadmium exerts its toxic effects in liver, providing valuable insights for targeted interventions and potential therapeutic strategies against acute hepatotoxicity (Li et al., 2023).

Plant-synthesized silver nanoparticles (Ag NPs) display diverse therapeutic properties, including antimicrobial, diabetic, anti-cancer, hepatoprotective, analgesic, anti-inflammatory, and antioxidant effects (Barhoum et al., 2016; Rehan et al., 2017). Ag NPs have been synthesized using various plant extracts such as turmeric (Alsammarraie et al., 2018), Aloe vera (Tippayawat et al., 2016), green tea (Parvathalu et al., 2023), neem (Verma and Mehata, 2016), basil (Ahmad et al., 2010), cinnamon (Premkumar et al., 2018), ginger (Mehata, 2021; Said et al., 2021), garlic (Rastogi and Arunachalam, 2011), onion (Khalilzadeh and Borzoo, 2016), Artemisia herba-alba A (Belaiche et al., 2021), eucalyptus, and Phoenix dactylifera L (Laouini et al., 2021). Helianthemum lippii, a medicinal plant rich in phytochemicals, offers promising potential for nanoparticle synthesis due to its therapeutic properties (Plescia et al., 2022). Its phytochemical constituents, including flavonoids, phenolic acids, tannins, and alkaloids, exhibit antioxidant, anti-inflammatory, and anticancer effects. Furthermore, H. lippii extracts contain phenolic compounds such as quercetin, chlorogenic acid, gallic acid, caffeic acid, and p-coumaric acid, as observed in a study (Ibtissam and Djahra, 2022).

Bioactive compounds in H. lippii extracts effectively synthesize Ag NPs, acting as both reducing and stabilizing agents, ensuring biocompatibility and therapeutic efficacy (Said et al., 2024). Using *Helianthemum lippii* phytochemicals for NP synthesis shows promise for targeted drug delivery, enhanced bioavailability, and reduced cytotoxicity, making *H. lippii*-based NPs attractive for treating various ailments, including heavy metal-induced hepatotoxicity. Despite their potential, more studies are needed due to concerns about prolonged or high-dose Ag NP exposure, emphasizing the importance of understanding Ag NP biodistribution and clearance (Saravanan et al., 2018; Shanmuganathan et al., 2018; Laib et al., 2023). Addressing Ag NPs’ biocompatibility and stability in physiological conditions is crucial for safe therapeutic use, although the lack of standardized protocols poses challenges (Suriyakalaa et al., 2013; Pugazhendhi et al., 2018; Ibtissam and Boutlilis, 2023; Zaater et al., 2024). Balancing the promise of Ag NPs for therapeutic applications with their limitations is essential for responsible field advancement and successful translation into clinical practice.

The aim of this study was to investigate the therapeutic efficacy of Ag NPs synthesized with a *H. lippii* extract for alleviating cadmium chloride (CdCl_2_)-induced hepatotoxicity. The primary objectives of this research were a comprehensive examination of biochemical parameters (enzymes, oxidative stress) and histopathology in Wistar rats exposed to sub-chronic CdCl_2_-induced toxicity. The novelty of this study concerns the methodology employed to synthesize Ag NPs using a natural extract of *H. lippii.* This distinctive approach is in line with environmentally friendly practices and also taps into the potential synergistic effects of plant-derived compounds and Ag NPs to limit hepatotoxicity. The advantages of this methodology lie in its sustainable nature, reduced ecological impact, and potential enhancement of therapeutic outcomes (Jeevanandam et al., 2022). This approach is in line with the global shift towards sustainable and eco-friendly methodologies in scientific research. By highlighting the importance of *H. lippii*, this study advances our understanding of innovative therapeutic strategies and paves the way for harnessing the inherent benefits of plant extracts for managing heavy metal-induced hepatotoxicity.

## 2 Experimental

### 2.1 Materials

Cadmium chloride (CdCl_2,_ 99%), silver nitrate (AgNO_3,_ 99.9%), potassium dihydrogen phosphate (KH_2_PO_4_, 99.5%), dibasic potassium phosphate (K_2_HPO_4,_ *99.95%*), ascorbic acid (vitamin C, 99.9%), ethylenediaminetetraacetic acid (EDTA, 99.0%), 2-thiobarbituric acid (TBA, 97.0%), salicylic acid (C₇H₆O₃, 95.5%), 5,5′-dithiobis-2-nitrobenzoic acid (DTNB, 98.28%), 4-nitro blue tetrazolium chloride (NBT, 99.9%), hydrogen peroxide (H_2_O_2_, 99.9%), ethanolamine (C_2_H_7_NO, 99%), o-cresolphthalein (C_22_H_18_O_4_, 95%), 8-hydroxyquinoline (C_9_H_7_NO, 99.99%), sodium potassium tartrate (KNaC_4_H_4_O_6_.4H_2_O, 99%), sodium iodide (NaI, 99%) potassium iodide (KI, 99.99%), copper sulphate (CuSO₄,98%), sodium hydroxide (NaOH 99.99%), imidazole (C_3_H_4_N_2_, 99%), pyruvic acid (C_3_H_4_O_3_, 99%), Tris pH 7.8 (C_4_H_11_NO_3_, 99.9%), L-alanine (C_3_H_7_NO_2_, 99%), diethanolamine pH 10.4 (C_4_H_11_NO_2_, 90%), chloroform (CHCl_3_, 94%) and magnesium chloride (MgCl_2_, 98%) were from Sigma-Aldrich.

### 2.2 Synthesis and characterization of Ag NPs from *Helianthemum lippii* extract

*Helianthemum lippii* aerial parts were collected in the Elhamadin region of the El-Oued province (Southeast Algeria), during the flowering season in March 2020. *Helianthemum lippii* was identified by Professor Atef Chouikh from the Faculty of Natural Science and Life at El Oued University. Approximately 10 g of the collected aerial parts was used for the preparation of the aqueous extract. The plant material was incubated in 100 mL of distilled water in the absence of light for 24 h. Following filtration with filter paper, the resulting extract was employed as reducing and capping agent for Ag NP synthesis. Their green synthesis was initiated by dissolving AgNO_3_ (1 mM) in 90 mL of distilled water, and the solution was kept in the dark to prevent photochemical reactions. A typical synthesis involved combining 10 mL of *H. lippii* extract with the AgNO_3_ solution. Ag NP formation was indicated by the appearance of a brownish hue. The reaction mixtures were then incubated in the dark at 60°C for 24 h. The resulting dried Ag NPs were prepared for subsequent experimental use and characterization (Laib et al., 2023).

UV–vis spectrophotometry (Shimadzu UV-1800 UV–Vis spectrophotometer, Japan) was used to determine the light absorbance of Ag NPs within the wavelength range of 200–800 nm. To assess Ag NP crystallinity and crystal structure, X-ray diffraction (XRD; PROTO AXRD Benchtop) was performed with CuKα radiation (30 kV and 20 mA) at a wavelength of 0.154281 Å, and a scanning speed of 0.05°. Ag NP size and morphology were examined using scanning electron microscopy (SEM; Thermo Scientific, Quatro, Themo Fisher Scientific, Germany) and their elemental composition was assessed by energy-dispersive X-ray (EDX) analysis.

### 2.3 Animal procurement and housing

Twenty-nine adult male Wistar albino rats, with a mean weight of 232.36 ± 3.81 g, were sourced from the Pasteur Institute animal facility in Alger, Algeria. Rats were housed at the Department of Molecular and Cellular Biology, University of El-Oued, Algeria, in a controlled laboratory environment (relative humidity of 64%, room temperature of 19°C, and 12 h of light and 24 h of darkness) for 2 weeks. Throughout the study, rats had *ad libitum* access to standard rat diet and tap water. Animal experiments and the experimental protocols were approved by the Institutional Animal Ethical Committee (IAEC), University of El Oued, Algeria.

### 2.4 Design of the cadmium toxicity experiment

Following acclimatization, the twenty rats were randomly divided into four groups, with each group consisting of five rats (*n* = 5). CdCl_2_ and Ag NPs doses were determined based on cadmium LD_50_ value and exposure time (Monsefi et al., 2010; Singh et al., 2018). The experimental groups were as follows:

Group I (control): Normal water consumption.

Group II: CdCl_2_ for 35 days in drinking water (50 mg/kg body weight/day)

Group III: Ag NPs (100 μg/kg, by intraperitoneal injection) for 35 days.

Group IV: CdCl_2_ exposure followed by Ag NPs (0.1 mg/kg body weight/day by intraperitoneal injection) for 15 days.

### 2.5 Ag NP sub-acute toxicity study

In accordance with the guidelines provided by the Organization for Economic Cooperation and Development (OECD) 425, a sub-acute toxicity study was conducted using nine rats, divided into three groups of male Wistar albino rats (*n* = 3/group). The control group (Group 1) received normal water and Groups 2 and 3 received Ag NPs (2 mg/kg and 10 mg/kg, respectively) by intraperitoneal injection. Then, various parameters, including signs of toxicity, body weight changes, adverse effects, movement, diarrhea, eye conditions and mortality, were closely monitored at different time points till day 14 in order to obtain insights into the sub-acute toxicity profile of the administered Ag NPs.

### 2.6 Body weight monitoring

Rats were weighed regularly in the different experimental groups. The mean body weight was calculated as follows:

Mean Body Weight = Sum of Individual Body Weights/Number of Rats.

Additionally, the body weight change (%) from baseline (initial body weight) was computed using the formula:

Body Weight Change (%) = (Final Body Weight—Initial Body Weight/Initial Body Weight) ×100.

### 2.7 Liver function biomarkers

Liver function was assessed by measuring key serum enzymes, including Asparate Transaminase (AST), Alanine Transaminase (ALT), Lactate Dehydrogenase (LDH), and Alkaline Phosphatase (ALP), and also serum glucose, albumin, calcium and total protein levels. After being sacrificed, rats under light anesthesia of chloroform via inhalation were decapitated as a method of euthanasia, following ethical guidelines and regulations. Blood was collected immediately afterwards by allowing it to flow from the severed neck, and the serum was separated. The serum was obtained after centrifuging the blood at a speed of 2,500 rpm for 10 min and was stored at −20°C until it was needed for biochemical analysis. The enzyme activity levels were determined using the relevant Spinreact kit (Barcelona, Spain) according to the manufacturer’s instructions.

### 2.8 Oxidative stress markers

To assess the oxidative stress levels in the liver, the concentration of Malondialdehyde (MDA), a widely recognized marker of lipid peroxidation and oxidative damage, was measured in liver extracts using a previously described method (Zaky et al., 2022). In addition, the liver antioxidant status was evaluated by measuring the concentration of reduced glutathione (GSH), using the method outlined by Kumar et al., (Kumar et al., 2021), and the activity of the key antioxidant enzymes catalase and Superoxide Dismutase (SOD), using the protocols established by Abu-El-Zahab et al., (Abu-El-Zahab et al., 2019), and Matović et al. (Matović et al., 2015), respectively. These enzymes play pivotal roles in the cell defense against oxidative stress by neutralizing reactive oxygen species (ROS).

### 2.9 Histology

Following sacrifice, rat liver tissues were immersed in a fixative solution, typically 10% formaldehyde, for time, to ensure the preservation of the tissue structure. Then, after gradual dehydration through a series of ethanol solutions, tissue samples were washed in toluene, a critical step to remove any residual ethanol, followed by paraffin embedding. Thin sections of 4_∼_6 µm were stained with hematoxylin (85%) and eosin (90%) (Sigma–Aldrich), a widely employed technique in histopathology to visualize cellular structures and identify abnormalities. The histopathological examination was conducted under a light microscope, allowing the detailed analysis of cellular architecture, tissue morphology, and the identification of any pathological changes induced by the experimental conditions.

### 2.10 Statistical analysis

Statistical analysis was conducted using Minitab (Version 13Fr) and Excel (version 2007). Data were expressed as mean ± standard error of the mean (SEM). Group comparisons were performed using the Student’s *t*-test. Images were processed and analyzed for standard error of the mean (SEM) using the ImageJ program.

## 3 Results and discussion

### 3.1 Characteristics of Ag NPs from *Helianthemum lippii* extract

**FIGURE 1:**
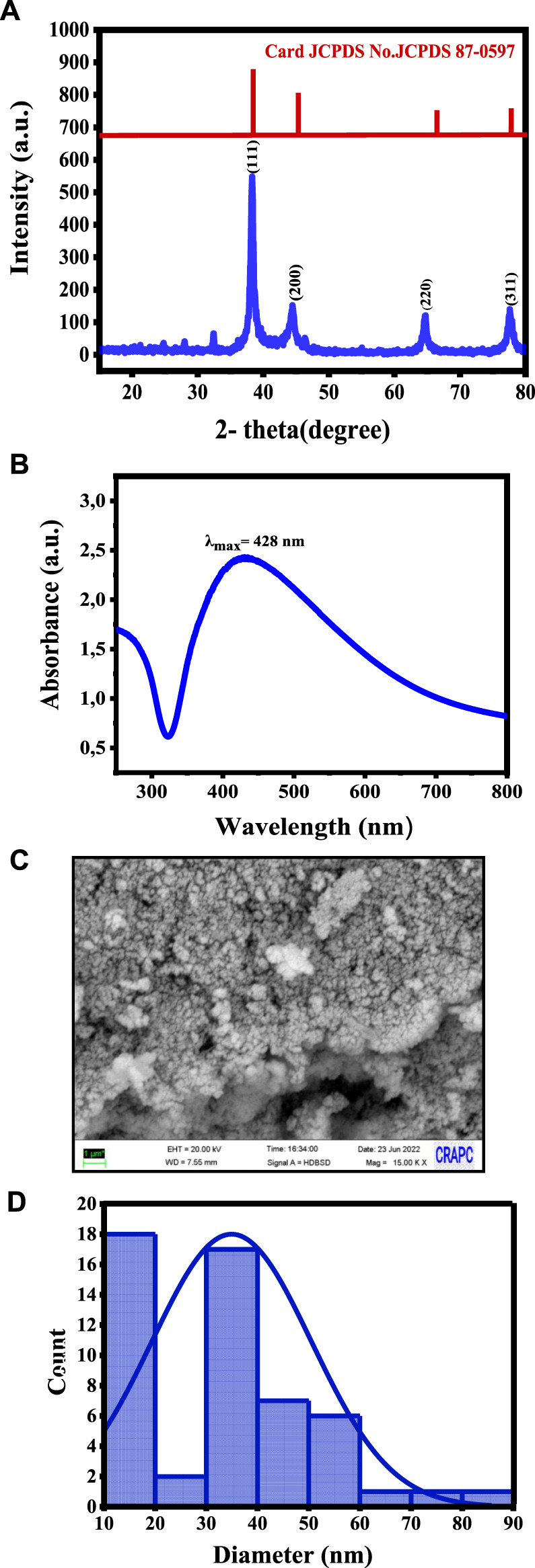
Characteristics of the synthesized Ag NPs: **(A)** XRD showing the crystalline structure, **(B)** UV-vis spectroscopy confirming the Ag NP formation, **(C)** SEM micrographs showing their morphology, and **(D)** Size distribution analysis.

The XRD pattern of the synthesized Ag NPs showed distinct diffraction peaks at 38.2°, 44.4°, 64.7°, and 77.5° (Figure 1A), corresponding to the (111), (200), (220), and (311) crystallographic planes of the face-centered cubic unit cell (Suganthi et al., 2019), respectively, in agreement with the characteristic peaks of metallic silver (JCPDS Card No. 04-0783) (Barhoum et al., 2015). The sharp and well-defined nature of these peaks indicates the high crystallinity of Ag NPs. The application of the Scherrer equation to the full width at half maximum of the peaks yielded a crystallite size of 12.84 nm, confirming the nano-scale dimensions and efficiency of the green synthesis approach.

UV-vis spectroscopy revealed a prominent surface plasmon resonance band at approximately 428 nm (Figure 1B), indicative of Ag NP presence. The absorption peak corresponded to the collective oscillation of free electrons in the Ag nanocrystals, resulting in a distinct color shift observed visually. Ag NP stability was attributed to biological functional groups from the *H. lippii* extract, acting as effective stabilizing agents. The UV-vis spectroscopy analysis provided information on the Ag NP size, confirming the successful green synthesis.

SEM showed that AG NPs had a sphere-like morphology with an average particle size of 35 ± 15 nm (Figure 1C), as determined by the particle size distribution analysis (Figure 1D). This indicated homogeneity and stability. SEM-EDX analysis confirmed the elemental composition, with a strong signal in the silver region and additional peaks corresponding to oxygen and carbon, suggesting their involvement as stabilizing agents or as residual components from the synthesis process.

### 3.2 Ag NP sub-acute toxicity study

For the sub-acute toxicity test of Ag NPs in Wistar albino rats, physiological parameters were monitored at various time points (0 h, 3 h, 24 h, 7 days, and 14 days) (Table 1). No rat died in both control and test groups (*n* = 3 animals/group), indicating the absence of acute toxic effects leading to mortality. All groups displayed normal movement, absence of diarrhea, and normal eye conditions throughout the study (“N” in Table 1). Altogether, these findings suggest the relative safety of Ag NPs at the doses tested in the sub-acute toxicity study (2 mg/kg and 10 mg/kg), supporting their potential for further investigation and application.

**TABLE 1:** Sub-acute toxicity assessment of Ag NP effect on physiological parameters in Wistar albino rats at different time points after intraperitoneal injection or not (Control) of 2 mg/kg and 10 mg/kg (Test; the two groups were merged in the table) of Ag NPs.

Parameters	0 h		3 h		24 h		7 days		14 days	
	Control	Test	Control	Test	Control	Test	Control	Test	Control	Test
**Dead rats**	0	0	0	0	0	0	0	0	0	0
**Movement**	N	N	N	N	N	N	N	N	N	N
**Diarrhoea**	N	N	N	N	N	N	N	N	N	N
**Eyes**	N	N	N	N	N	N	N	N	N	N

N, Normal

In the next stection, the effect of Ag NPs on cadmium-induced liver toxicity was assessed in adults Wistar albino rats. To this aim, rats were divided in four groups (*n* = 5/group): Group I (control; normal water); Group II (CdCl_2_ for 35 days in drinking water); Group III (Ag NPs by intraperitoneal injection for 35 days); and Group IV (CdCl_2_ exposure for 35 days followed by Ag NPs by intraperitoneal injection for 15 days).

### 3.3 Body weight changes

**FIGURE 2:**
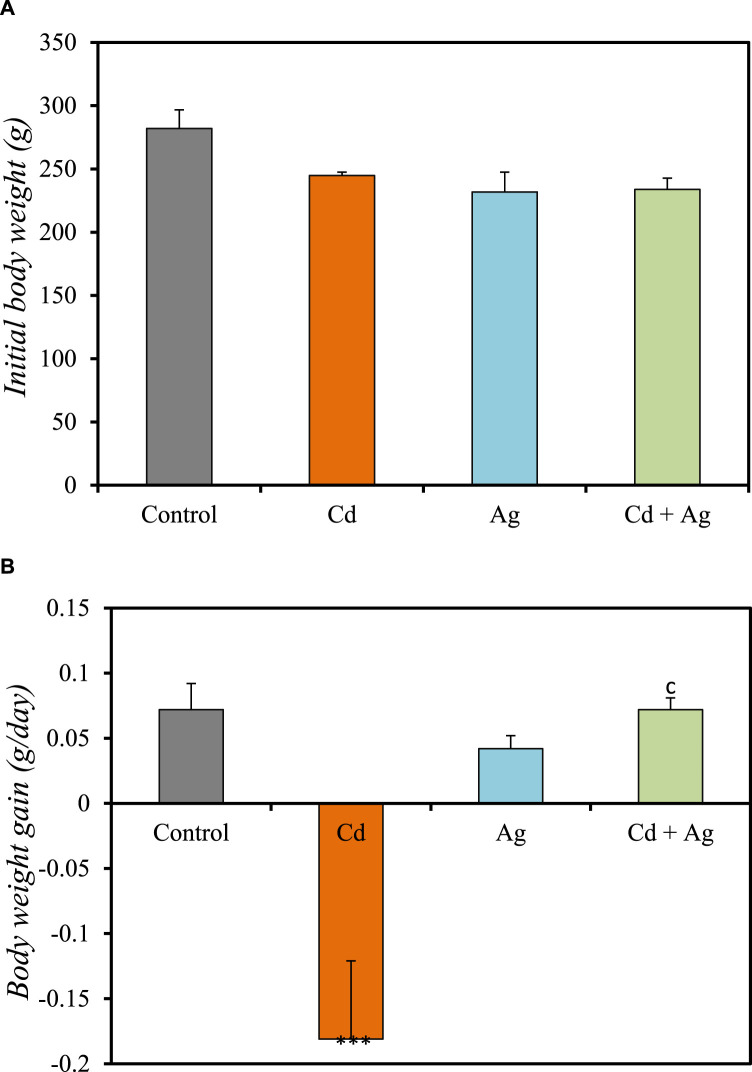
Comparison of the initial body weight **(A)** and body weight gain **(B)** in the four rat groups. Group I (Control): Normal water consumption. Group II (Cd): addition of CdCl_2_ (50 mg/kg body weight/day) in drinking water for 35 days. Group III (Ag): Ag NPs (100 μg/kg, intraperitoneal injection) for 35 days. Group IV (Cd + Ag): CdCl_2_ exposure (like for Group II) followed by Ag NPs (0.1 mg/kg, body weight/day by intraperitoneal injection) for 15 days ***p < 0.001 vs. Group (I) C *p* < 0.001: vs. Group II.

Figure 2 and Supplementary Table S1 (Supplementary Material) show the body weight-related parameters in the four groups. In the control group (Group I), the initial body weight was 282 ± 14.7 g and weight increased by 0.072 ± 0.02 g/day (Figures 2A,B). In Group II (CdCl_2_ exposure), the initial body weight (244.8 ± 2.69 g) decreased over time (−0.181 ± 0.06 g/day) (Figures 2A,B). In Group III (Ag NPs alone), the initial body weight was 231.8 ± 15.7 g and weight increased by 0.042 ± 0.017g/day (Figures 2A,B), further confirming their relative safety. In Group IV (CdCl_2_ + Ag NPs), the initial body weight was 233.8 ± 8.95 g and weight increased by 0.072 ± 0.009 g/day (Figures 2A,B), similarly to what observed in the control group. The negative effect of CdCl_2_ exposure on body weight is attributed to metabolic abnormalities induced by oxidative stress (Ali et al., 2021). Conversely, Ag NP administration after CdCl_2_ exposure improved body weight, emphasizing their potential protective effect against Cd toxicity, supported by their ability to permeate tissues and maintain a large surface area.

### 3.4 Biochemical parameters

**FIGURE 3:**
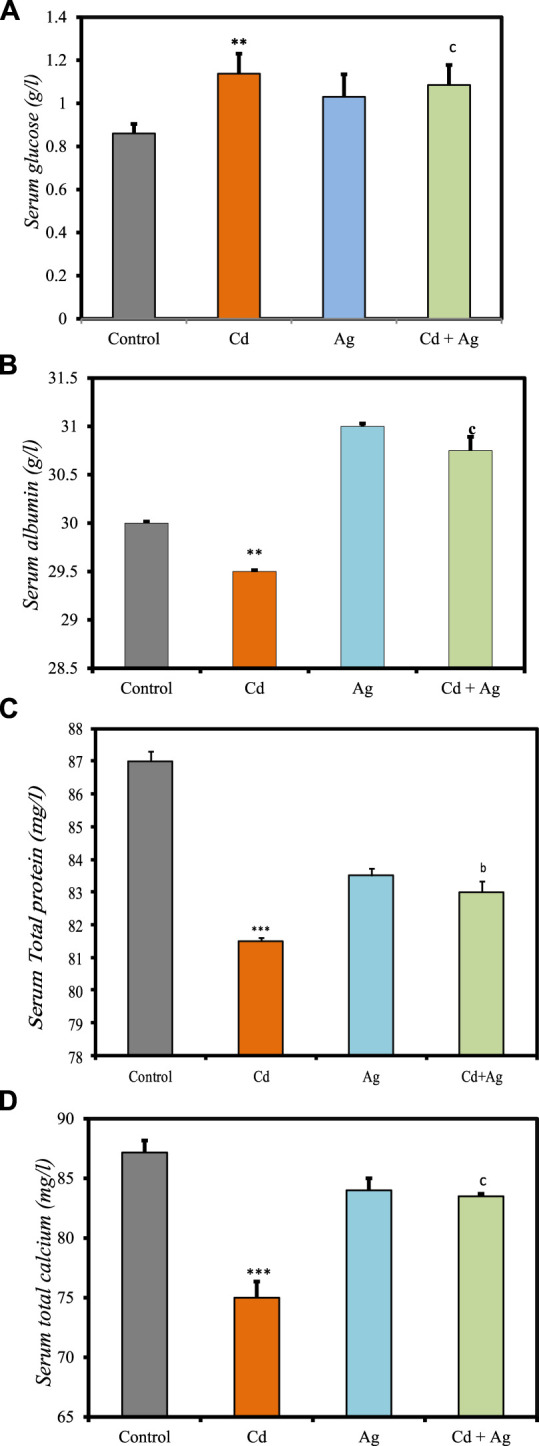
Comparison of serum biomarker levels in the four rat groups. Group I (Control): Normal water consumption. Group II (Cd): addition of CdCl_2_ (50 mg/kg body weight/day) in drinking water for 35 days. Group III (Ag): Ag NPs (100 μg/kg, intraperitoneal injection) for 35 days. Group IV (Cd + Ag): CdCl_2_ exposure (as in Group II) followed by Ag NPs (0.1 mg/kg, body weight/day by intraperitoneal injection) for 15 days. **(A)** Serum glucose **(B)** serum Albumin, **(C)** total protein, **(D)** serum total calcium. (inset) ***p < 0.001: significantly different from Group I), ^a^ *p* < 0.05, ^b^ *p* < 0.01, ^c^ *p* < 0.001: significantly different from Group II.

Figure 3 shows the impact of CdCl_2_ exposure and the potential protective effect of Ag NPs on various serum biochemical markers, as detailed in Supplementary Table S2 of the Supplementary Material. Rats exposed to CdCl_2_ (Group II) exhibited significantly higher serum glucose levels compared to the control group (1.13 ± 0.093 g/L vs 0.86 ± 0.044 g/L; *p* < 0.01) (Figure 3A), indicating altered glucose metabolism. Additionally, CdCl_2_ exposure led to a notable reduction in albumin (29.5 ± 0.0131 g/L) (Figure 3B) and serum total protein (81.5 ± 0.1 mg/L) (Figure 3C), implying potential hepatic damage affecting protein synthesis. Conversely, rats treated with Ag NPs alone (Group III) showed no significant differences in these parameters compared to the control group, suggesting that Ag NPs alone do not impact these markers. In Group IV, where Ag NPs were administered after CdCl_2_ exposure, serum glucose levels decreased (1.085 ± 0.09 g/L) compared to Group II. Furthermore, total protein (83 ± 0.34 mg/L) and albumin (30.75 ± 0.141 g/L) levels increased, although the difference was not significant. Serum total calcium levels were notably affected by the treatments, with CdCl_2_ exposure resulting in a significant decrease (75 ± 1.354 mg/L; *p* < 0.001) compared to the control (Figure 3D). However, treatment with Ag NPs stabilized serum calcium levels, showing a slight increase compared to the Cd group but remaining lower than the control (83.5 ± 0.2 mg/L; *p* < 0.01). These results suggest that Ag NPs may mitigate CdCl_2_-induced alterations in glucose metabolism and hepatic function, supporting their potential therapeutic role in combating Cd toxicity.

The observed rise in serum glucose in Cd-exposed rats (Group II) aligns with literature indicating potential damage to pancreatic β-cells, contributing to altered glucose metabolism (El Muayed et al., 2012). Reduced serum total protein and albumin levels in Group II suggest impaired protein synthesis, likely due to Cd-induced liver damage. Conversely, Ag NPs alone (Group III) did not significantly alter these parameters, indicating their lack of inherent toxicity. In Group IV, where Ag NPs were administered post-CdCl_2_ exposure, there was a notable improvement in serum glucose levels and a trend towards restored total protein and albumin levels compared to Group II. Previous studies by Rehman et al. (2023) and El-Baz et al. (2023) showed that Ag NPs improved protein and blood glucose levels, consistent with their anti-hyperglycemic, antioxidant, and anti-inflammatory actions. Ag NPs interact directly with target tissues like the liver, adipose tissue, and skeletal muscle, modulating lipid and glucose metabolism pathways. They may activate AMPK, PPARγ, and other pathways controlling lipid and glucose homeostasis, and modulate hormone activity (Zhang et al., 2019). These findings indicate a multifaceted mechanism of Ag NPs’ therapeutic effects, involving cellular signaling modulation and hormone secretion. Moreover, they suggest a potential mitigating effect of Ag NPs on CdCl_2_-induced alterations, supporting their therapeutic potential, as seen in previous research showing the preventive effects of Ag NPs on liver damage in diabetic mice (Wahab et al., 2022), highlighting their restorative capabilities amidst oxidative stress and hepatoprotection.

### 3.5 Liver function biomarkers

**FIGURE 4:**
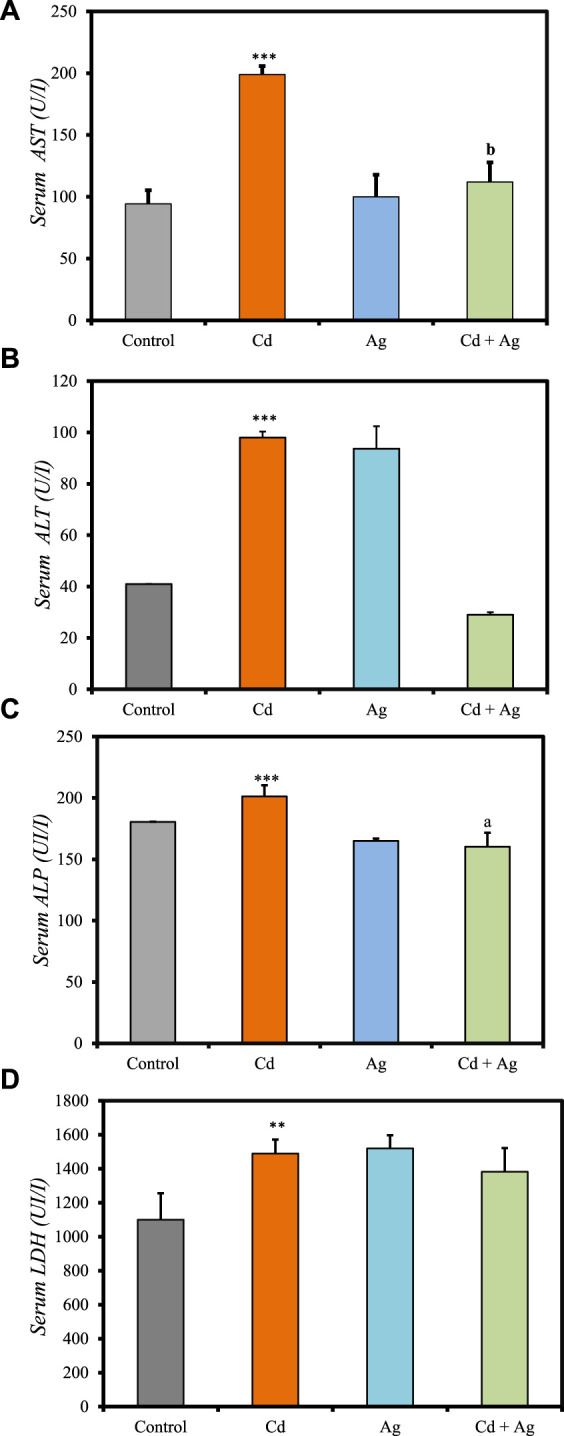
Comparison of liver function biomarker levels in the four rat groups. Group I (Control): Normal water consumption. Group II (Cd): addition of CdCl_2_ (50 mg/kg body weight/day) in drinking water for 35 days. Group III (Ag): Ag NPs (100 μg/kg, intraperitoneal injection) for 35 days. Group IV (Cd + Ag): CdCl_2_ exposure (as in Group II) followed by Ag NPs (0.1 mg/kg, body weight/day) for 15 days. **(A)** serum AST, **(B)** serum ALT, **(C)** serum ALP, **(D)** serum LDH. *p < 0.05; **p < 0.01; ***p < 0.001: significantly different from Group I. ^a^ *p* < 0.05; ^b^ *p* < 0.01; ^c^ *p* < 0.001 (*n* = 5 rats/group): significantly different from Group II.

Figure 4 summarizes the differences in liver function biomarkers in the four groups at the experiment end (Supplementary Table S3, Supplementary Material). In Group II (CdCl_2_-treated), serum AST (199.00 ± 6.80 U/L vs. 94.3 ± 20.9 in controls) (Figure 4A), ALT (98.00 ± 2.31 U/L vs. 41 ± 0.001) (Figure 4B), ALP (201.23 ± 9.2 U/L vs. 180.50 ± 0.19) (Figure 4C) and LDH (1,489.1 ± 82.6 U/L vs. 1,100 ± 156) (Figure 4D), levels were significantly increased compared with the control group (Group I), indicating substantial hepatotoxicity induced by CdCl_2_ exposure. Conversely, in Group III (Ag NPs), their levels were similar to those in the control group: AST = 100.0 ± 17.9 U/L, ALT = 93.67 ± 8.75 U/L, LDH = 1,519.7 ± 77.2 U/L, and ALP = 164.99 ± 1.94 U/L. This suggests that Ag NPs alone do not induce significant hepatic alterations, reinforcing their biocompatibility in normal conditions. In Group IV (CdCl_2_ + Ag NPs), AST, ALT, LDH, and ALP increase was limited compared with the CdCl_2_-treated group: AST = 112 ± 15.8 U/L, ALT = 29.00 ± 0.0001 U/L, LDH = 1,382 ± 140 U/L, and ALP = 160.3 ± 11.3 U/L. This indicates that Ag NPs may mitigate CdCl2-induced hepatic damage through their antioxidant and anti-inflammatory properties, as evidenced by various *in vitro* and *in vivo* studies (Arozal et al., 2022). They scavenge free radicals, neutralize ROS, enhance antioxidant enzyme activity, and suppress inflammatory pathways, thereby reducing oxidative stress and inflammation in the liver. Moreover, Ag NPs exhibit metal chelating capabilities, binding to heavy metals like Cd and facilitating their excretion from the body, thereby reducing their toxicity (Zhang et al., 2019). This prevents heavy metal accumulation in liver tissues and supports hepatic function. Furthermore, Ag NPs may enhance the activity of endogenous metal detoxification enzymes, such as metallothioneins, and modulate hepatic metabolism by regulating detoxification pathways (El-Baz et al., 2023). These nanoparticles can enhance phase I and phase II detoxification enzyme activities, essential for metabolizing and eliminating heavy metals and other xenobiotics (Arozal et al., 2022; Kocak et al., 2022).

### 3.6 Oxidative stress markers in liver

**FIGURE 5:**
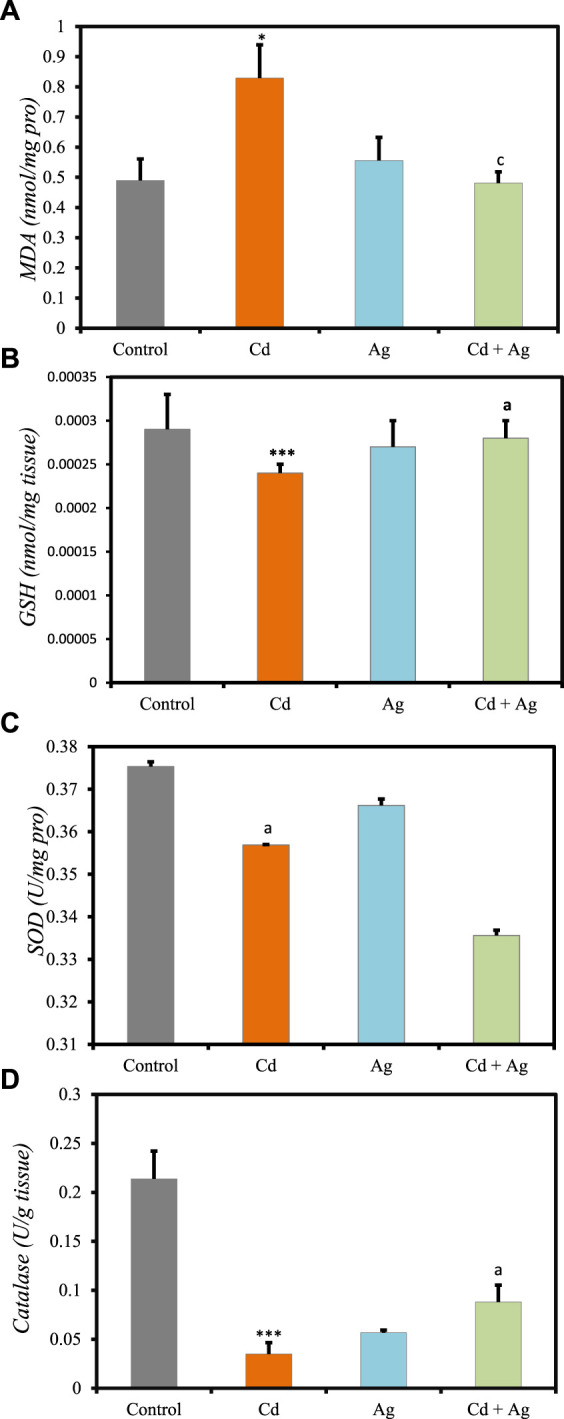
Comparison oxidative stress biomarker levels in the four rat groups. Group I (Control): Normal water consumption. Group II (Cd): addition of CdCl_2_ (50 mg/kg body weight/day) in drinking water for 35 days. Group III (Ag): Ag NPs (100 μg/kg, intraperitoneal injection) for 35 days. Group IV (Cd + Ag): CdCl_2_ exposure (as in Group II) followed by Ag NPs (0.1 mg/kg, body weight/day by intraperitoneal injection) for 15 days. **(A)** MDA, **(B)** GSH, **(C)** SOD, **(D)** Catalase. *p < 0.05; ***p < 0.001: significantly different from Group I. ^a^ *p* < 0.05; ^c^ *p* < 0.001, (*n* = 5 rats/group): significantly different from Group II.

Comparison of liver oxidative stress markers among the four groups (Figure 5, Supplementary Table S4, Supplementary Material) revealed significant differences. Rats exposed to CdCl_2_ (Group II) exhibited markedly increased levels of MDA compared to the control (Group I) (0.829 ± 0.11 nmol/mg pro vs 0.4894 ± 0.07) (Figure 5A), indicating elevated oxidative stress and potential liver damage. Additionally, antioxidant defenses were significantly diminished in Group II compared to Group I: reduced GSH levels = 0.00024 ± 0.10 nmol/g tissue vs 0.00029 ± 0.00004 (Figure 5B). Furthermore, SOD activity was reduced in Group II (0.35 ± 0.001 U/mg pro) compared to Group I (0.37 ± 0.001) (Figure 5C), and catalase activity was notably decreased (0.034 ± 0.011 U/g tissue vs 0.2137 ± 0.028) (Figure 5D). Conversely, rats treated with Ag NPs alone (Group III) showed no significant differences in these parameters compared to Group I: MDA = 0.5557 ± 0.077 nmol/mg pro, GSH = 0.00027 ± 0.00003 nmol/g tissue, SOD = 0.36 ± 0.001 U/mg pro, and catalase = 0.056 ± 0.002 U/g tissue. In rats treated with Ag NPs after CdCl_2_-exposure (Group IV), oxidative stress markers were reduced, and antioxidant defenses were improved compared to Group II. Specifically, MDA concentration was reduced (0.4809 ± 0.037 nmol/mg protein), while GSH (0.0002 ± 0.00002 nmol/g tissue) and catalase (0.088 ± 0.017 U/g tissue) were increased, although SOD levels remained unchanged (0.33 ± 0.001 U/mg pro) compared to Group II. These results suggest that Ag NPs exert a protective effect, potentially mitigating liver damage caused by cadmium-induced toxicity.

Previous studies indicated that Ag NPs exhibit robust antioxidant properties by scavenging reactive oxygen species (ROS) like superoxide anion (O^2-^), hydrogen peroxide (H_2_O_2_), and hydroxyl radical (OH^•^), thereby reducing oxidative stress and preventing damage to biomolecules (Veeramani and Shanmugam, 2023). They activate antioxidant response pathways, such as the nuclear factor erythroid 2-related factor 2 (Nrf2) pathway, promoting the expression of antioxidant and detoxification genes. Ag NPs stimulate Nrf2 signalling, leading to the transcriptional activation of genes encoding key antioxidants like SOD, CAT, GPx, and HO^−1^ (Veeramani and Shanmugam, 2023). Moreover, they modulate intracellular signaling pathways such as mitogen-activated protein kinase (MAPK) and phosphoinositide 3-kinase/protein kinase B (PI3K/Akt), which regulate cell survival and antioxidant gene expression (Salama et al., 2023). These combined actions enhance cellular antioxidant defenses and confer cytoprotective effects against oxidative stress-induced damage. Consequently, Ag NPs hold promise for therapeutic applications in conditions associated with oxidative stress, including those induced by Cd-exposure.

These data indicate that oxidative stress was increased in Group II, consistent with literature data on cadmium-induced hepatic injury (Seif et al., 2019). However, this effect was significantly limited in exposed rats treated with Ag NPs (Group IV), showcasing their protective effect by stabilizing membranes, limiting oxidative stress, and addressing inflammatory processes. CdCl_2_ is an oxidative stressor, leading to increased ROS production and subsequent depletion of endogenous antioxidants, resulting in peroxidative damage of the liver cell membranes (Asagba et al., 2002). The sub-chronic exposure to CdCl_2_ in this study induced oxidative stress, depleting the antioxidant system and increasing MDA levels. These findings can be explained by impairment of the enzymatic antioxidant defense system, particularly GSH, as indicated by its reduction in Group II.

### 3.7 Histological alterations in liver

**FIGURE 6:**
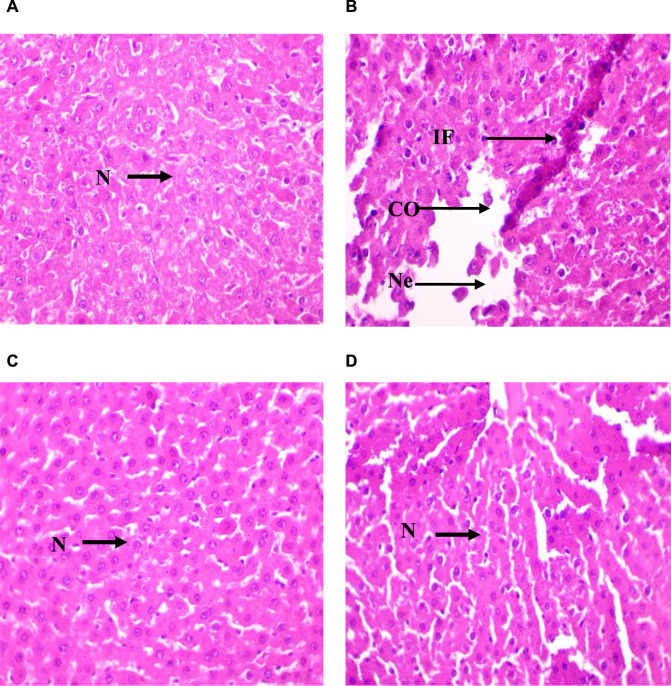
Micrographs of representative rat liver sections from the different experimental groups showing the effect of CdCl_2_ exposure and the mitigating effect of Ag NPs. **(A)**: Liver section of a rat from Group I (control); **(B)** Liver section of a rat from Group II (CdCl_2_ exposure); **(C)** Liver section from a rat of Group III (Ag NPs); **(D)** Liver section of rat from Group IV (Ag NPs after CdCl_2_ exposure). N; normal hepatocyte, IF; Inflammatory cells, CO; Congestion of sinusoids, NE; Necrosis; × 40.

The semi-quantitative analysis of the architectural damage in the liver from rats of the four groups (Figure 6 and Supplementary Table S5, Supplementary Material) showed the presence in Group II (CdCl_2_ exposure) of portal fibrosis (++), inflammatory cell infiltration (+++), liver necrosis (+), sinusoid congestion and dilation (+++), hepatic vein dilation and congestion (+++), and cytoplasmic vacuolations (+) (Figure 6B). This suggests severe hepatic damage induced by CdCl_2_ exposure. In Group III (Ag NPs alone), liver architecture was not affected, indicating that Ag NPs alone did not induce significant histopathological alterations (Figure 6C). In Group IV (Ag NPs after CdCl_2_ exposure), architectural damage parameters were improved compared with Group II (Figure 6D). Inflammatory cell infiltration and hepatic vein dilation and congestion were still present, albeit reduced compared with Group II. Conversely, portal fibrosis, liver necrosis, sinusoid congestion and dilation, and cytoplasmic vacuolations were not detected. This suggests that Ag NPs mitigate CdCl_2_-induced histopathological alterations and supports their potential therapeutic role in alleviating liver damage caused by CdCl_2_ exposure.

These findings suggest that Ag NPs mitigate CdCl_2_-induced histopathological alterations by stabilizing mitochondrial membrane potential, inhibiting caspase activation, and promoting DNA repair mechanisms, thus preventing Cd-induced cell death and supporting liver tissue integrity (Das et al., 2023). Additionally, Ag NPs enhance liver regeneration and repair processes by promoting hepatocyte proliferation, angiogenesis, and extracellular matrix remodeling. They may also modulate growth factors and cytokines involved in liver regeneration, such as hepatocyte growth factor (HGF) and transforming growth factor-beta (TGF-β), further supporting tissue repair mechanisms (Das et al., 2023; Salama et al., 2023). These combined effects underscore the potential therapeutic applications of Ag NPs in mitigating Cd-induced hepatotoxicity and preserving liver function.

### 3.8 Evaluation in the context of prior research

Ag NPs synthesized without plant extracts have intrinsic properties (e.g., antioxidant, anti-inflammatory, and antimicrobial effects) that contribute to mitigate hepatotoxicity. These properties enable Ag NPs to neutralize ROS, reduce inflammation, control microbial infections, and promote tissue repair following damage. The use of green synthesis methods to optimize biocompatibility enhances their applicability for hepatotoxicity treatment. However, concerns persist about Ag NP safety, particularly after prolonged or high-dose exposure, that can be influenced by various factors, such NP size, shape, coating, and administration route. Achieving a balance between therapeutic benefits and safety requires rigorous research and regulatory scrutiny. Challenges, such as biodistribution, clearance, antibiotic resistance, biocompatibility, stability and the lack of standardized protocols, underscore the need of international collaboration to ensure reliable and reproducible Ag NP use in therapeutic settings.

Ag NP synthesis using extracts from plants, such as *H. lippii,* is a green and eco-friendly approach. This method reduces the reliance on conventional chemical methods and uses bioactive compounds from plants as reducing and stabilizing agents during Ag NP synthesis. These plant-mediated Ag NPs display enhanced biocompatibility, reduced cytotoxicity, and potential therapeutic properties, making them suitable for biomedical applications. In the context of hepatotoxicity mitigation, these Ag NPs exhibit antioxidant and anti-inflammatory properties, effectively limiting liver oxidative stress and inflammatory responses. The use of plant extracts is in line with green nanotechnology principles, promoting sustainability and accessibility, and offering a promising avenue for the development of safer and more effective treatments for liver-related disorders.

El-Houseiny et al. investigated the impact of Ag NPs synthesized in the presence of a *Moringa oleifera* extract on *A. hydrophila*-infected Nile tilapia (*Oreochromis niloticus*) (El-Houseiny et al., 2021). *Aeromonas hydrophila* infection led to elevated levels of biochemical markers and inflammatory biomarkers, genotoxic effects, and tissue damage. However, exposure to Ag NPs at different concentrations significantly improved these parameters and reduced mortality at 0.8 mg/L. This suggests that Ag NPs, synthesized through green methods, offer a non-cytotoxic alternative to mitigate the detrimental effects of A*. hydrophila* infection in Nile tilapia. Nauroze et al. explored the effect of *N. sativa*-conjugated Ag NPs in mice presenting hexavalent chromium-induced hepatotoxicity and nephrotoxicity by monitoring organ indices, serum biochemical markers, oxidative stress parameters, and histopathological changes (Nauroze et al., 2023). Hexavalent chromium exposure led to increased liver index and serum levels of ALT, AST, ALP, MDA, and creatinine and to decreased total protein levels. Histopathological analysis showed hepatic and renal damage. Administration of *Nigella sativa* Ag NPs mitigated the oxidative damage induced by hexavalent chromium, suggesting a potential protective role against chromium-induced toxicity. Metwally et al. (Metwally et al., 2021) studied in mice the protective effect of Ag NPs biosynthesized with *Salvia officinalis* leaf extract on hepatic tissue injury induced by *P. chabaudi*, a protozoan parasite that cause malaria. Both pre-treatment and post-treatment with Ag NPs resulted in substantial parasitemia reduction compared with the untreated infected group. They investigated the antiplasmodial and hepatoprotective effects of *S. officinalis* leaf extract-biosynthesized Ag NPs, particularly *Plasmodium chabaudi*-induced inflammation and hepatic oxidative stress markers. Lalsangpuii et al. (Lalsangpuii et al., 2022) synthesized Ag NPs using a *Spilanthes acmella* leaf extract. In mice harboring Dalton’s lymphoma ascites tumors, co-administration of Ag NPs and doxorubicin reduced doxorubicin-induced toxicity by decreasing the serum toxicity markers and enhancing antioxidant activities. The biosynthesized Ag NPs exhibited superior free-radical scavenging activities compared with the leaf extract, indicating their potential biomedical applications. Hassan et al. (Hassan et al., 2023) explored the potential of Ag NPs decorated with curcumin (Ag NP-Cur) to increase metformin efficacy in diabetic rats, specifically targeting drug-induced hepatotoxicity. Rats receiving the combined treatment displayed significant improvement in glucose concentration, lipid profile, liver function, toxicity markers, and redox indicators compared with untreated diabetic controls. This combination reduced oxidative stress, toxic burden, and inflammation. The histological analysis and comet assay results supported these findings, indicating restored cellular structure and nuclear DNA. The authors concluded that Ag NP-Cur enhances metformin anti-diabetic effects by mitigating drug-induced hepatotoxicity through alleviating oxidative stress and inflammation (Hassan et al., 2023). Alamri et al. (Alamri et al., 2022) investigated the hepatoprotective effects of vanillic acid, silymarin, and vanillic acid-loaded Ag NPs in male rats with CCl_4_-induced hepatotoxicity. Vanillic acid-loaded Ag NPs and silymarin significantly protected rats, normalizing biochemical markers and liver tissue appearance, suggesting the therapeutic potential of vanillic acid-loaded Ag NPs in mitigating CCl_4_-induced hepatotoxicity.

Although our study primarily addresses the immediate effects of Ag NPs in alleviating Cd-induced hepatotoxicity, we acknowledge the necessity for subsequent research to ascertain the enduring efficacy and safety of these treatments. Further investigations should aim to evaluate the persistence of Ag NPs’ therapeutic benefits over prolonged durations and examine any potential adverse effects associated with extended exposure. Additionally, longitudinal studies monitoring the health status of treated subjects over time would offer valuable insights into the long-term efficacy and safety profile of Ag NP interventions. Incorporating such long-term assessments and follow-up studies is crucial for gaining a comprehensive understanding of the therapeutic potential and safety considerations of Ag NPs in combating heavy metal-induced toxicity.

## 4 Conclusion

This study investigated the potential therapeutic efficacy of Ag NPs synthesized from *H. lippii* extract in mitigating Cd-induced hepatotoxicity in 20 adult Wistar rats (*n* = 5/group), divided into one control (untreated/unexposed) and three experimental groups (Ag NPs alone, exposure to CdCl_2_ only for 35 days, and exposure to CdCl_2_ for 35 days followed by Ag NPs for 15 days). The comprehensive analyses of biochemical markers, oxidative stress parameters, histopathological features, and body weight changes indicated that treatment with Ag NPs after CdCl_2_-exposure significantly improved many of these parameters that were altered by CdCl_2_. This indicates that Ag NP administration effectively attenuated the adverse effects of CdCl_2_ exposure on liver function, as evidenced by the restoration of liver enzyme levels. Additionally, Ag NPs exhibited antioxidative properties by enhancing SOD, catalase and hepatic glutathione activities. This antioxidant capacity contributed to the mitigation of oxidative stress induced by CdCl_2_ exposure, as reflected by the reduced MDA levels. The histopathological analysis further supported the protective role of Ag NPs as indicated by the improved liver tissue architecture and decreased inflammatory response induced by CdCl_2_. Notably, the CdCl_2_ and Ag NP-treated group displayed signs of recovery, reduced vacuolization, fatty tissue degradation, and inflammatory cell infiltration. This was associated with body weight gain. Altogether, these findings highlight the potential benefits of Ag NPs in counteracting the adverse effects of exposure to CdCl_2_. They also suggest that Ag NPs, synthesized from *H. lippii* extract, hold promise as a therapeutic strategy to alleviate CdCl_2_-induced hepatotoxicity. However, more studies are needed to elucidate the underlying mechanisms and optimize Ag NP dose/regimens for safe and effective therapeutic applications. This study contributes valuable insights into the potential of Ag NPs as a protective agent against heavy metal toxicity, emphasizing the importance of research in this burgeoning field.

## Data Availability

The original contributions presented in the study are included in the article/Supplementary Material, further inquiries can be directed to the corresponding author.
