# Supplementary material for: Therapeutic potential of silver nanoparticles from Helianthemum lippii extract for mitigating cadmium-induced hepatotoxicity: liver function parameters, oxidative stress, and histopathology in wistar rats
Source: Front Bioeng Biotechnol. 2024 Jun 27;12:1400542. doi: 10.3389/fbioe.2024.1400542 (PMC11240457; doi:10.3389/fbioe.2024.1400542)
Supplement: Supplementary file 1 [file Table1.DOCX]

**Therapeutic Potential of Silver Nanoparticles from *Helianthemum lippii* Extract for Mitigating Cadmium-Induced Hepatotoxicity: Liver Function Parameters, Oxidative Stress, and Histopathology in Wistar Rats**

Ibtissam LAIB ^1, 2,3^, Boutlilis DJAHRA ALI^1,3^, Ali Alsalme,^4^ Mikhael Bechelany^5,6^, Ahmed Barhoum^7*^

^1^ Department of Cellular and Molecular Biology, Faculty of Natural and Life Sciences, El Oued University, El Oued 39000, El Oued, Algeria

^2^ Higher School of Saharan Agriculture, El Oued, Algeria

^3^ Laboratory of Biology, Environment and Health, Faculty of Natural and Life Sciences, El Oued University, El-Oued 39000, Algeria

^4^ Department of Chemistry, College of Science, King Saud University, Riyadh, 11451, Saudi Arabia

^5^Institut Européen des Membranes, IEM, UMR-5635, Univ Montpellier, ENSCM, CNRS, Place Eugene Bataillon, 34095 Montpellier, France

^6^ Gulf University for Science and Technology, GUST, Kuwait

^7^ NanoStruc Research Group, Chemistry Department, Faculty of Science, Helwan University, Cairo, 11795, Egypt

*Corresponding author: [ahmed.barhoum@science.helwan.edu.eg](mailto:ahmed.barhoum@science.helwan.edu.eg)

**Supplementary information**

Table S1. Initial body weight and body weight gain at the study end. Group I (Control): Drinking water; Group II (Cd): addition of CdCl_2_ (50 mg/kg body weight/day) in drinking water for 35 days; Group III (Ag): Ag NPs (100 μg/kg, intraperitoneal injection) for 35 days; Group IV (Cd + Ag): CdCl_2_ treatment as in Group II followed by Ag NPs (0.1 mg/kg body weight/day, intraperitoneal injection) for 15 days. ***p < 0.001 vs Group I; c p < 0.001 vs Group II (n = 5 rats/group).

| Rat  groups | Group I  (Control) | Group II  (Cd) | Group III  (Ag) | Group IV  (Cd + Ag) |
| --- | --- | --- | --- | --- |
| Parameters | Drinking water | CdCl_2_ in drinking water for 35 days | Ag NPs by intraperitoneal injection  for 15 days | Ag NPs after exposure to CdCl_2_ |
| Initial body weight (g) | 282.0 ± 14.7 | 244.8 ± 2.69 | 231.8 ± 15.7 | 233.8 ± 8.95 |
| Body weight gain (g/d) | 0.072 ± 0.020 | -0.181 ± 0.06*** | 0.042 ± 0.017 | 0.072 ± 0.009^c^ |

Table S2. Serum biochemical marker levels in the different rat groups at the study end. Group I (Control): Drinking water; Group II (Cd): addition of CdCl_2_ (50 mg/kg body weight/day) in drinking water for 35 days; Group III (Ag): Ag NPs (100 μg/kg body weight/day, intraperitoneal injection) for 35 days; Group IV (Cd + Ag): CdCl_2_ treatment as in Group II followed by Ag NPs (0.1 mg/kg, body weight/day, intraperitoneal injection) for 15 days. **p < 0.01, ***p < 0.001 vs Group I; b p < 0.01, c p < 0.001 vs Group II (n = 5 rats/group).

| Rat groups | Serum glucose (g/l) | Serum albumin (g/l) | Serum total protein (mg/l) | Serum total calcium (mg/l) |
| --- | --- | --- | --- | --- |
| Group I (Control) | 0.86±0.044 | 30±0.016 | 87±0.3 | 87.17±1.0002 |
| Group II (Cd) | 1.13±0.093^**^ | 29.5±0.0131^**^ | 81.5±0.1^***^ | 75±1.354^***^ |
| Group III (Ag) | 1.03±0.105 | 31±0.0289 | 83.5±0.224 | 84±1.01 |
| Group IV (Cd + Ag) | 1.085±0.09^c^ | 30.75±0.141^c^ | 83±0.34^b^ | 83.5±0.2^c^ |

Table S3. Serum markers of liver function in rat groups. Group I (Control): Drinking water; Group II (Cd): addition of CdCl_2_ (50 mg/kg body weight/day) in drinking water for 35 days; Group III (Ag): Ag NPs (100 μg/kg body weight/day, intraperitoneal injection) for 35 days; Group IV (Cd + Ag): CdCl_2_ treatment as in Group II followed by Ag NPs (0.1 mg/kg body weight/day, intraperitoneal injection) for 15 days. ** p < 0.01; *** p < 0.001 vs Group I; a p < 0.05; b p < 0.01 vs Group II (n = 5 rats/group).

| Rat Groups | Serum TGO (U/l) | Serum TGP (U/I) | Serum LDH (UI/I) | Serum ALP (UI/I) |
| --- | --- | --- | --- | --- |
| Group I (Control) | 94.3 ± 20.9 | 41 ± 0.001 | 1100 ± 156 | 180.50 ± 0.19 |
| Group II (Cd) | 199.00 ± 6.80  *** | 98.00 ± 2.31  *** | 1489.1 ± 82.6  ** | 201.23 ± 9.2  *** |
| Group III (Ag) | 100.0 ± 17.9 | 93.67 ± 8.75 | 1519.7 ± 77.2 | 164.99 ± 1.94 |
| Group IV (Cd+ Ag) | 112 ± 15.8^b^ | 29.00 ± 0.0001 | 1382 ± 140 | 160.3 ± 11.3^a^ |

Table S4. Liver oxidative stress markers in the four rat groups. Group I (Control): Drinking water; Group II (Cd): CdCl_2_ (50 mg/kg body weight/day) in drinking water for 35 days; Group III (Ag): Ag NPs (100 μg/kg body weight/day, intraperitoneal injection) for 35 days; Group IV (Cd + Ag): CdCl_2_ treatment as in Group II followed by Ag NPs (0.1 mg/kg body weight/day, intraperitoneal injection) for 15 days. * p <0.05, *** p <0.001 vs Group I; a p <0.05, c p <0.001 vs Group II (n = 5 rats/group).

| Rat Groups | MDA (nmol/mg pro) | SOD (U/mg pro) | GSH (nmol/mg tissue) | Catalase (U/g tissue) |
| --- | --- | --- | --- | --- |
| Group I (Control) | 0.4894± 0.07 | 0.37±0.001 | 0.00029± 0.00004 | 0.2137± 0.028 |
| Group II (Cd) | 0.829± 0.11^*^ | 0.35± 0.001^*^ | 0.00024± 0.0001^***^ | 0.034± 0.01^***^ |
| Group III (Ag) | 0.5557± 0.077 | 0.36± 0.001 | 0.00027± 0.00003 | 0.056± 0.002 |
| Group IV (Cd + Ag) | 0.4809± 0.037^c^ | 0.33± 0.001 | 0.0002± 0.00002^a^ | 0.088± 0.017^a^ |

Table 6: Semi-quantitative results of the histological analysis of liver architecture damage in the different rat groups: Group I (Control): Drinking water; Group II (Cd): addition of CdCl_2_ (50 mg/kg body weight/day) in drinking water for 35 days; Group III (Ag): Ag NPs (100 μg/kg body weight/day, intraperitoneal injection) for 35 days; Group IV (Cd + Ag): CdCl_2_ treatment as in Group II followed by Ag NPs (0.1 mg/kg body weight/day, intraperitoneal injection) for 15 days (n = 5 rats).

| Rat groups | Group I | Group II | Group III | Group IV |
| --- | --- | --- | --- | --- |
| Parameters | Control | Cd | Ag | Cd + Ag |
| Portal fibrosis | - | ++ | - | - |
| Inflammatory infiltration | - | +++ | - | + |
| Hepatic necrosis | - | + | - | - |
| Sinusoid congestion and dilation | - | +++ | - | + |
| Hepatic vein dilation and congestion | - | +++ | - | - |
| Cytoplasmic vacuolations | - | + | - | - |
